# Supplementary material for: Increased iron uptake by splenic hematopoietic stem cells promotes TET2-dependent erythroid regeneration
Source: Nat Commun. 2024 Jan 15;15:538. doi: 10.1038/s41467-024-44718-0 (PMC10789814; doi:10.1038/s41467-024-44718-0)
Supplement: Supplementary file 1 — Supplementary Information [file 41467_2024_44718_MOESM1_ESM.pdf]

Supplementary information

Increased iron uptake by splenic hematopoietic stem cells promotes TET2-dependent erythroid regeneration

Yu-Jung Tseng[1,8], Yuki Kageyama[2,8], Rebecca L. Murdaugh[3], Ayumi Kitano[2], Jong Hwan Kim[4], Kevin A. Hoegenauer[2], Jonathan Tiessen[3], Mackenzie H. Smith[2], Hidetaka Uryu[5,6], Koichi Takahashi[5,6], James F. Martin[4,7], Md Abul Hassan Samee[4], and Daisuke Nakada[1,2,3*]

[1]Graduate Program in Translational Biology and Molecular Medicine, Baylor College of Medicine, Houston, TX 77030
[2]Department of Molecular and Human Genetics, Baylor College of Medicine, Houston, TX 77030
[3]Graduate Program in Developmental Biology, Baylor College of Medicine, Houston, TX 77030
[4]Department of Integrative Physiology, Baylor College of Medicine, Houston, TX 77030
[5]Department of Leukemia, The University of Texas MD Anderson Cancer Center, Houston, TX 77030
[6]Department of Genomic Medicine, The University of Texas MD Anderson Cancer Center, Houston, TX 77030
[7]Cardiomyocyte Renewal Laboratory, Texas Heart Institute, Houston, TX 77030

[8]These authors contributed equally: Yu-Jung Tseng, Yuki Kageyama

[*]Correspondence: Daisuke Nakada, One Baylor Plaza, R713, Houston TX 77030, United States; email: nakada@bcm.edu; phone: 713-798-1175

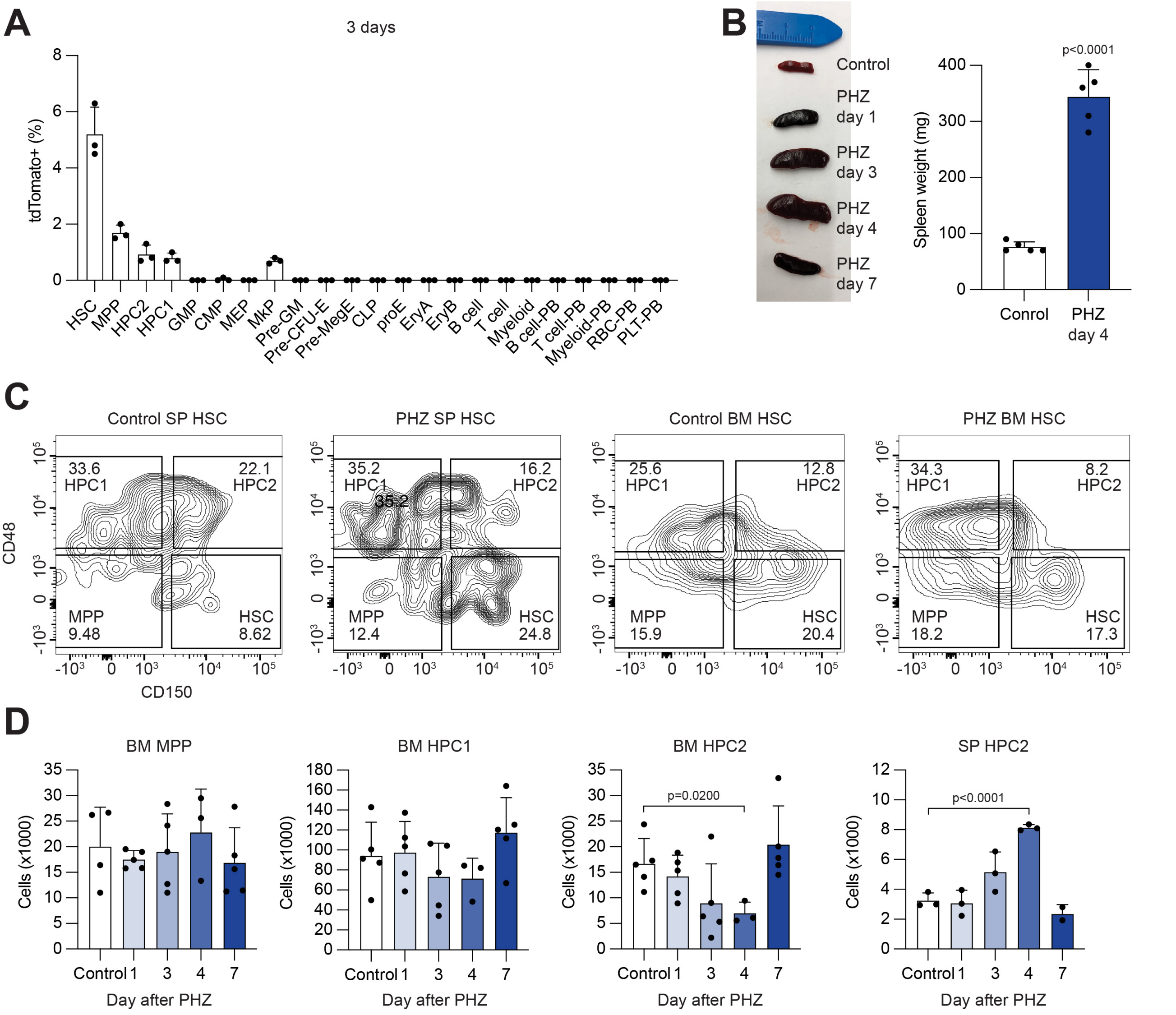

Supplementary Figure 1. The impact of PHZ-induced hemolytic anemia on the hematopoietic system.

(**A**) Lineage tracing with the Krt18-tdT strain at an early time point (3 days after the first tamoxifen injection, n=3).

(**B**) Images of spleens collected from control or PHZ-treated mice at day 1, 3, 4 and 7 post treatment. Spleen weight on day 4 of PHZ treatment is shown in the right panel (n=5).

(**C**) Representative flow cytometry plots of CD150 and CD48 expression in control or PHZ-treated whole bone marrow cells (BM) and splenocytes (SP). Cells were gated on LSK cells.

(**D**) The total numbers of bone marrow MPP, HPC1, HPC2, and splenic HPC2 at the indicated time after PHZ treatment (n=5, 5, 5, 3, and 5 for control, days 1, 3, 4, and 7, respectively).

Comparisons were performed by unpaired, two-tailed Student's t-test. All data represent mean±standard deviation. All numbers (n) are independent animals. Source data are provided as a Source Data file.

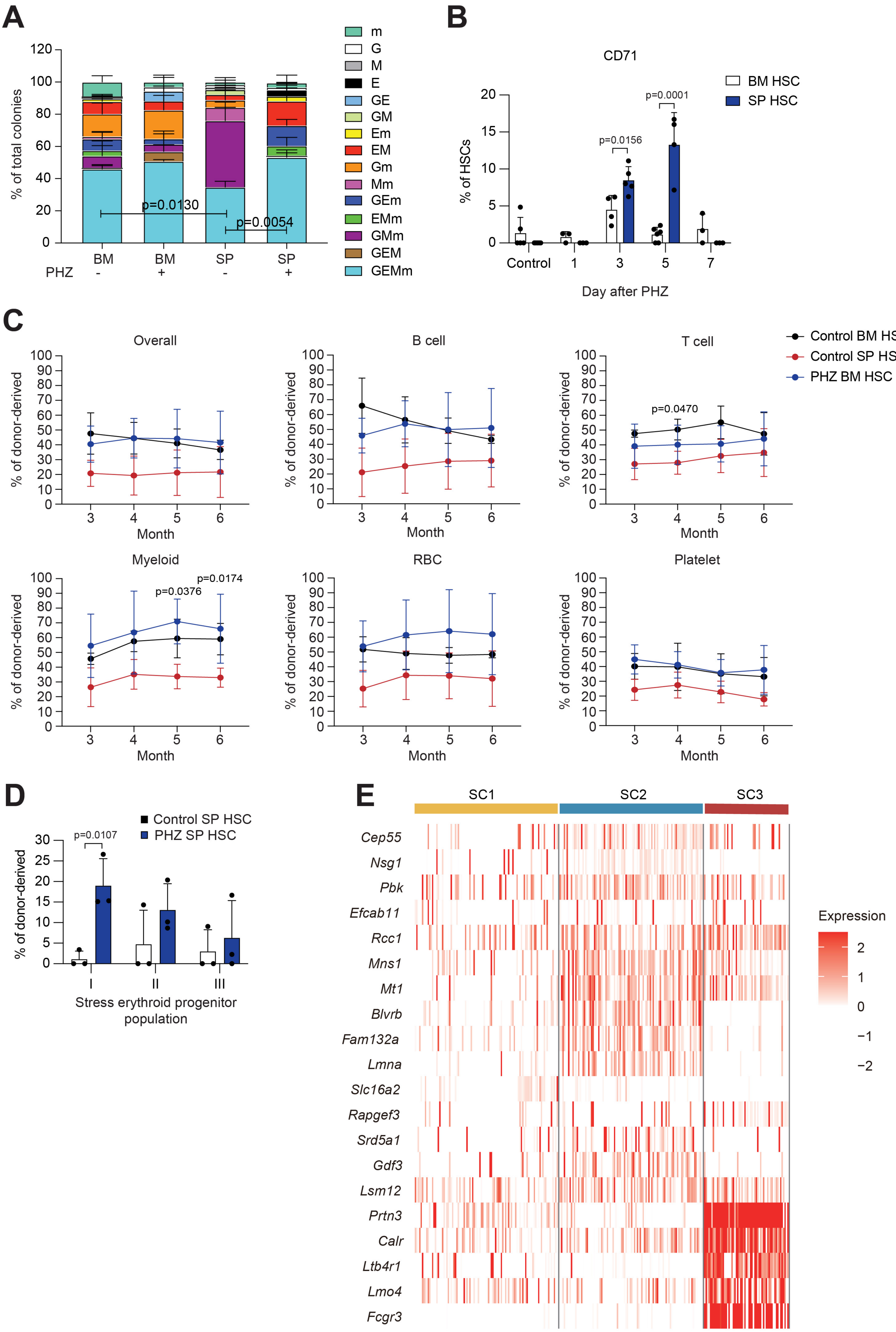
A
% of total colonies
120
100
80
60
40
20
0
p=0.0130
p=0.0054
BM
BM
SP
SP
PHZ
-
+
-
+
m
G
M
E
GE
GM
Em
EM
Gm
Mm
GEm
EMm
GMm
GEM
GEMm
B
CD71
% of HSCs
20
15
10
5
0
p=0.0156
p=0.0001
BM HSC
SP HSC
Control
1
3
5
7
Day after PHZ
C
Overall
B cell
T cell
Control BM HSC
Control SP HSC
PHZ BM HSC
% of donor-derived
p=0.0470
Month
Myeloid
RBC
Platelet
p=0.0376
p=0.0174
D
Control SP HSC
PHZ SP HSC
% of donor-derived
p=0.0107
I
II
III
Stress erythroid progenitor population
E
SC1
SC2
SC3
Cep55
Nsg1
Pbk
Efcab11
Rcc1
Mns1
Mt1
Blvrb
Fam132a
Lmna
Slc16a2
Rapgef3
Srd5a1
Gdf3
Lsm12
Prtn3
Calr
Ltb4r1
Lmo4
Fcgr3
Expression
2
1
0
−1
−2

Supplementary Figure 2. PHZ-induced hemolytic anemia alters the potential of splenic HSCs.
(**A**) Colony forming assays with single-cell sorted bone marrow or splenic HSCs from either control or PHZ-treated mice and scored for different colonies identities (n=3). G; granulocytes, E; erythrocytes, M; megakaryocytes, m; macrophages/monocytes.
(**B**) Frequency of CD71 positive fraction of control or PHZ-treated bone marrow and splenic HSCs (n=5, 3, 5, 6, and 3 for control, days 1, 3, 5, and 7, respectively).
(**C**) Transplantation of 50 control splenic HSCs or bone marrow HSCs from mice treated or untreated with PHZ. Results of control spleen HSCs are from Figure 2. Overall reconstitution and those of 5 blood lineages were analyzed at 3-6 months post transplantation (n=2, 4, and 3 for untreated bone marrow, untreated spleen, and PHZ-treated bone marrow, respectively).
(**D**) Frequency of donor-derived stress erythroid population I-III in the spleen of recipient mice transplanted with either control splenic HSCs or PHZ-treated splenic HSCs (n=3).
(**E**) Heatmap of differentially expressed genes within clusters SC1, SC2, and SC3 (n=4).
Comparisons were performed by unpaired, two-tailed Student’s t-test. All data represent mean±standard deviation. All numbers (n) are independent animals. Source data are provided as a Source Data file.

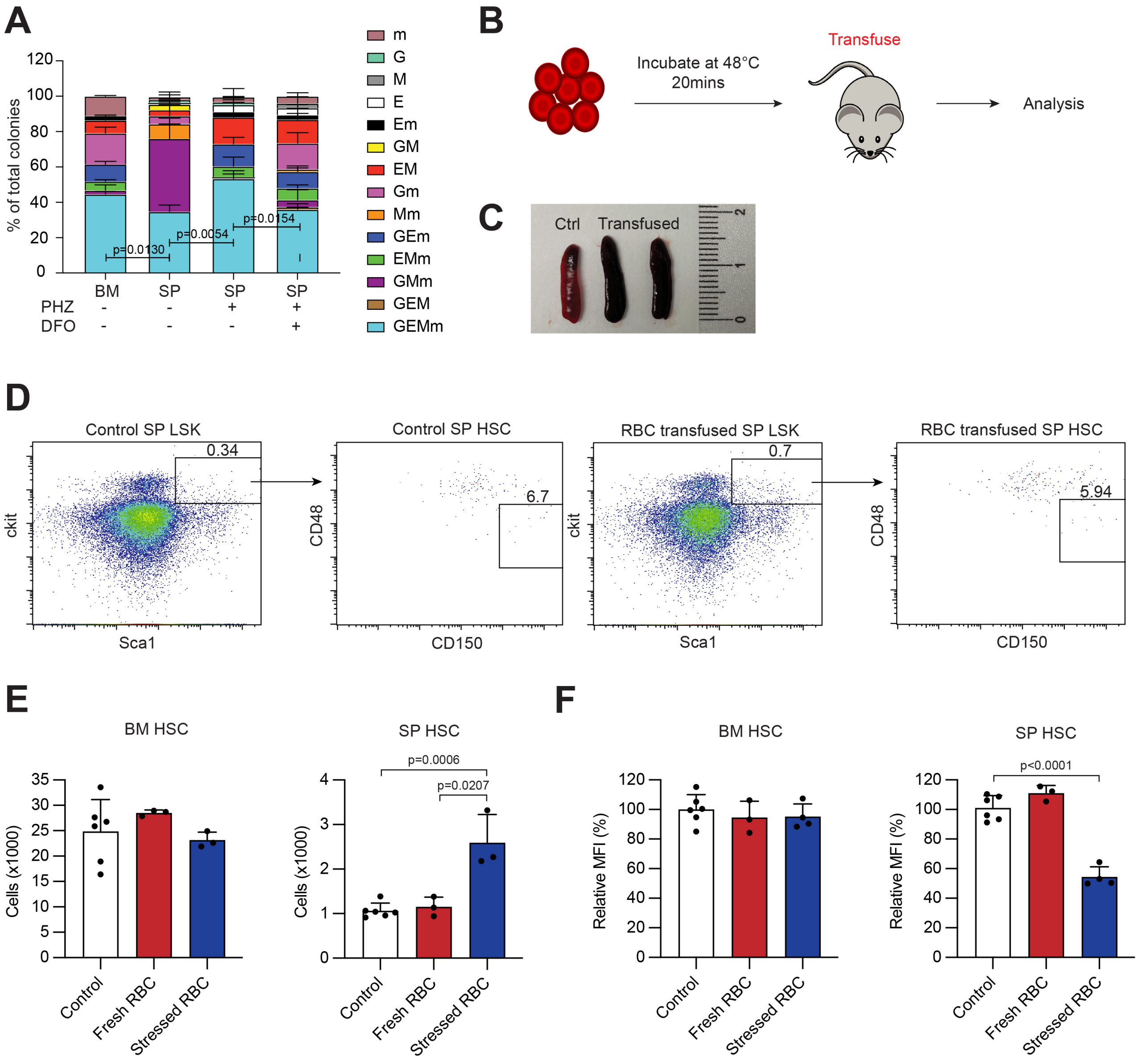

Supplementary Figure 3. Increased iron uptake in splenic HSCs after transfusion of stressed erythrocytes.

(**A**) Colony forming assays with single cell sorted bone marrow or splenic HSCs from mice with the indicated treatments (n=3).

(**B**) Schematic diagram of stressed erythrocyte transfusion.

(**C**) An image of spleens collected from mice untreated or transfused with stressed erythrocytes.

(**D**) Representative flow cytometry plots of control or stressed erythrocyte-transfused splenic LSK and HSCs.

(**E**) Total numbers of bone marrow (left) or splenic (right) HSCs of mice untreated or transfused with fresh or stressed erythrocytes (n=6, 3, and 3 for untreated, fresh erythrocytes-transfused, and stressed erythrocytes-transfused, respectively).

(**F**) Relative changes of calcein AM MFI in bone marrow (left) and splenic (right) HSC of mice untreated or transfused with fresh or stressed erythrocytes (n=6, 3, and 4 for untreated, fresh erythrocytes-transfused, and stressed erythrocytes-transfused, respectively).

Comparisons were performed by unpaired, two-tailed Student's t-test. All data represent mean±standard deviation. All numbers (n) are independent animals. Source data are provided as a Source Data file. Mouse and red blood cell illustrations were adapted from Creazilla (https://creazilla.com/nodes/18581-big-eared-grey-mouse-clipart, https://creazilla.com/nodes/1990678-erythrocyte-clipart) under the Creative Commons license CC0.

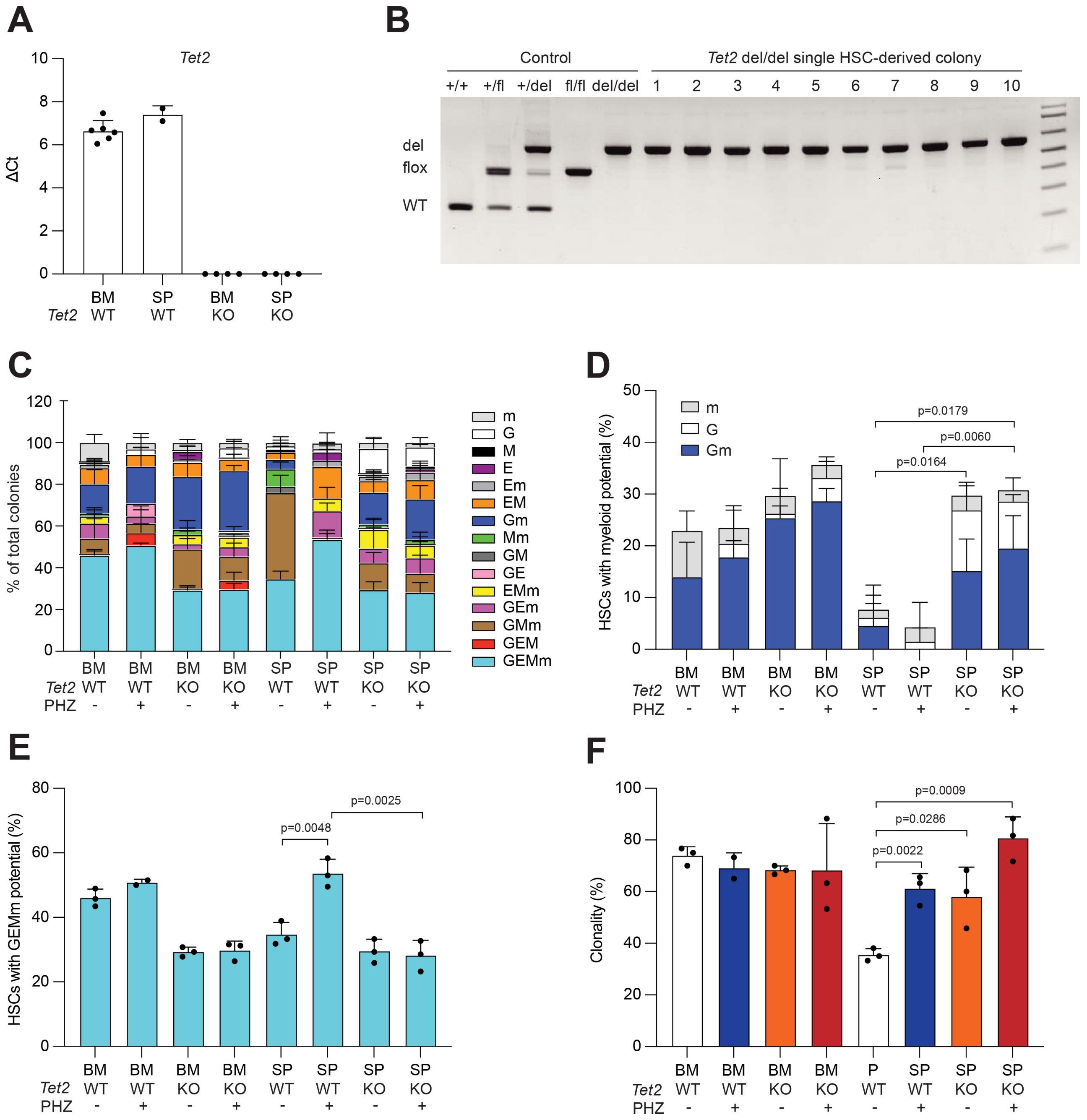

Supplementary Figure 4. PHZ-induced increase in the erythroid potential of splenic HSCs is dependent on TET2.

(**A**) Quantitative PCR analysis of *Tet2* mRNA expression in control or *Tet2* knockout bone marrow or splenic HSCs (n=6, 2, 4, and 4 for wild-type bone marrow, wild-type spleen, *Tet2* knockout bone marrow, and *Tet2* knockout spleen, respectively). Shown are ΔCt values of *Tet2* relative to *Actb*.

(**B**) Genomic PCR of the *Tet2* loci in single HSC-derived colonies from poly (I:C) treated *Mx1-Cre; Tet2*$^{fl/fl}$ mouse (*Tet2* del/del single HSC-derived colony). An amplicon from tail DNA of wild-type mouse (+/+), untreated *Mx1-Cre; Tet2*$^{+/fl}$ mouse (+/fl), poly (I:C) treated *Mx1-Cre; Tet2*$^{+/fl}$ mouse (+/del), untreated *Mx1-Cre; Tet2*$^{fl/fl}$ mouse (fl/fl), and poly (I:C) treated *Mx1-Cre; Tet2*$^{fl/fl}$ mouse (del/del) are shown as control.

(**C-F**) Colony forming assays with single-cell sorted bone marrow or splenic HSCs from wild-type or *Tet2* knockout mice with or without PHZ treatment. Frequency of colonies containing myeloid potential (**D**), frequency of GEMm colonies (**E**), and clonality (**F**) are shown (n=3).
Comparisons were performed by unpaired, two-tailed Student's t-test. All data represent mean±standard deviation. All numbers (n) are independent animals. Source data are provided as a Source Data file.

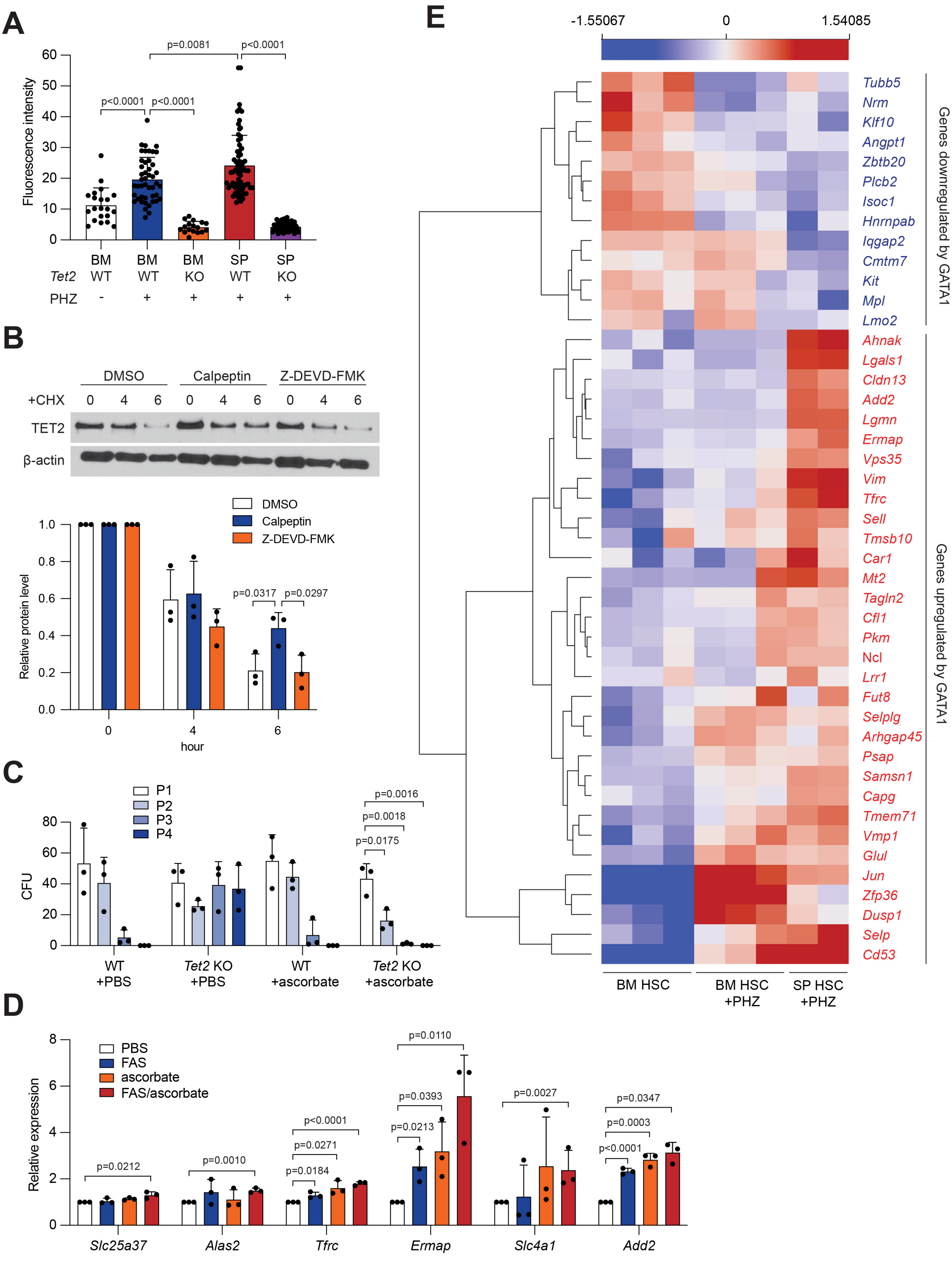

Supplementary Figure 5. TET2 protein levels in erythroid progenitors and the expression of GATA1-regulated genes in HSCs.

(**A**) TET2 protein levels in ProE and CFU-E cells isolated from mice with the indicated genotypes and treatments assessed by immunofluorescence staining (n=21, 46, 17, 70, and 55 cells for untreated wild-type bone marrow, PHZ-treated wild-type bone marrow, PHZ-treated *Tet2* knockout bone marrow, PHZ-treated wild-type spleen, and PHZ-treated treated *Tet2* knockout spleen, respectively).
(**B**) Representative Western blot analysis (upper) and quantification (lower) of TET2 levels in LSK cells treated with DMSO, calpeptin, and Z-DEVD-FMK in the presence of cycloheximide for 0, 4, and 6 hours (n=3).
(**C**) Colony-forming assay using whole bone marrow cells from wild-type or Tet2 knockout mice. Cells were replated for four passages in methylcellulose media with or without ascorbate supplementation (n=3)
(**D**) Relative expression of genes involved in erythropoiesis or iron metabolism in HSCs cultured with FAS, ascorbate, and FAS plus ascorbate. Data is presented as relative $2^{-\Delta\Delta Ct}$ compared to control PBS (n=3).
(**E**) GATA1-regulated genes were extracted from Tanimura et al. by comparing the transcriptomes of G1E-ER-GATA1 cells treated with or without E2, yielding 4,658 genes also discovered in our RNA-seq. 45 out of 4,658 GATA1 regulated genes were differentially expressed in PHZ-treated splenic HSCs compared to untreated bone marrow HSCs. Genes repressed or induced by GATA1 induction in Tanimura et al. are labeled in blue or red texts, respectively.
Comparisons were performed by unpaired, two-tailed Student's t-test. All data represent mean±standard deviation. Numbers (n) are cells from one animal per group in (**A**) and independent animals in (**B,C,D**). Source data are provided as a Source Data file.

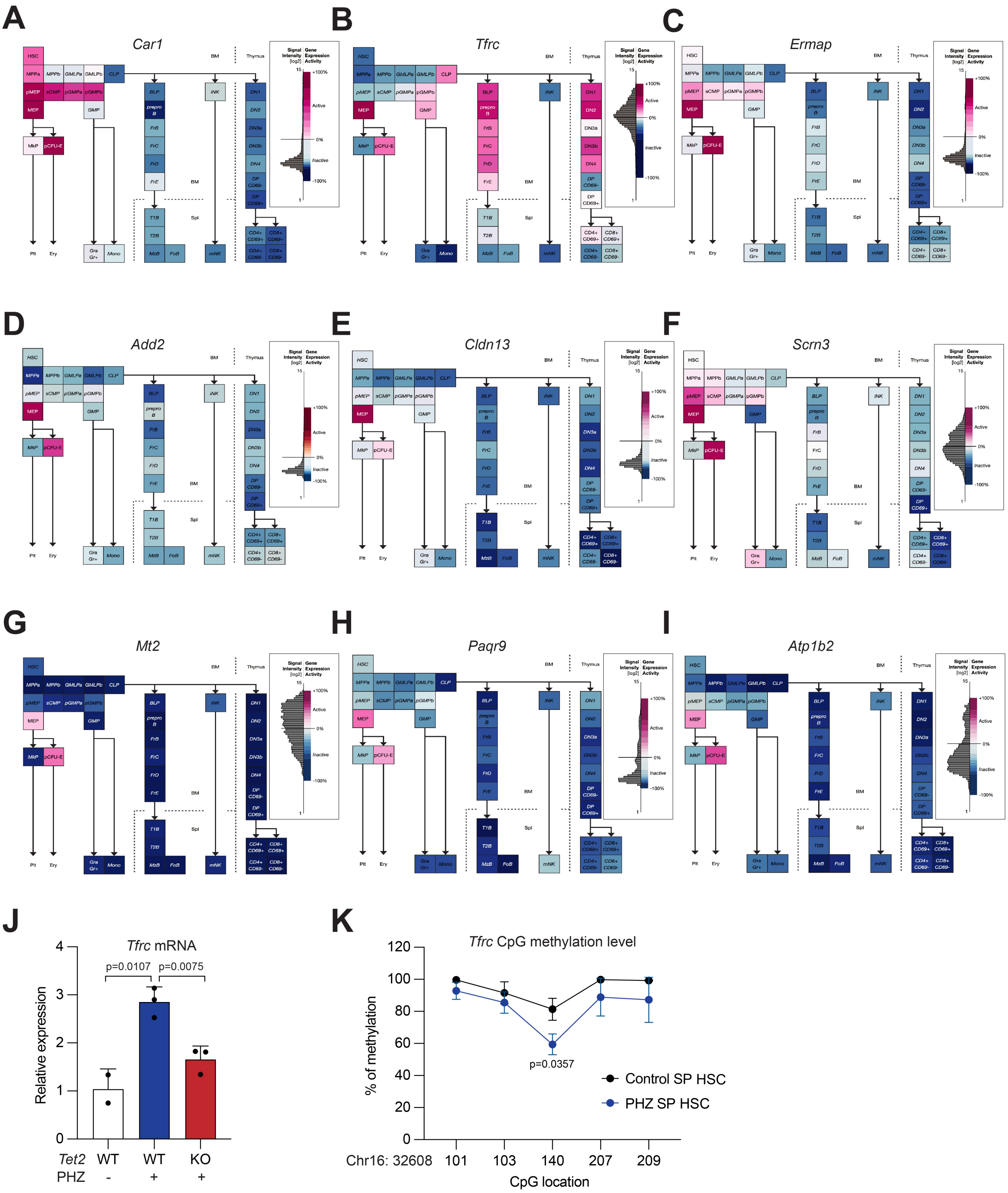

Supplementary Figure 6. RNA-seq reveals the induction of erythroid-related genes in splenic HSCs after PHZ treatment.

(**A-I**) Expression patterns of 9 erythroid-biased genes among the top 30 most differentially upregulated genes in PHZ-treated splenic HSCs, based on the Gene Expression Commons. The ranges of signal intensity and gene expression activity are shown in the inlets.

(**J**) Relative expression of *Tfrc* mRNA in WT or *Tet2* KO splenic HSCs with or without PHZ treatment. Data is presented as relative $2^{-\Delta\Delta Ct}$ compared to control splenic HSC (n=2, 3, 3 for untreated wild-type, PHZ-treated wild-type, and PHZ-treated *Tet2* knockout, respectively).
(**K**) Bisulfite high-throughput amplicon-seq of *Tfrc* methylation level at the indicated CpG sites in splenic HSCs with or without PHZ treatment (n=2 for untreated spleen and n=3 for PHZ-treated spleen).
Comparisons were performed by unpaired, two-tailed Student's t-test. All data represent mean±standard deviation. All numbers (n) are independent animals. Source data are provided as a Source Data file.

Supplementary Table 1

REAGENT or RESOURCE	SOURCE	IDENTIFIER	DILUTION
Antibodies			
TET2 (D6C7K) Rabbit mAb (Mouse Specific)	Cell Signaling Technology	Cat# 36449S	1:500, 1:1000
DyLight™ 554 Phalloidin	Cell Signaling Technology	Cat# 13054S	1:200
Goat anti-Rabbit IgG Secondary Antibody, Alexa Fluor 488	Invitrogen	Cat# A-11008	1:500
PE-Cy5 anti-mouse CD150	BioLegend	Cat# 115912	1:100
PE anti-mouse CD48	BioLegend	Cat# 103406	1:100
PE-Cy7 anti-mouse CD48	BioLegend	Cat# 103424	1:100
APC anti-mouse Sca-1	eBioscience	Cat# 17-5981-82	1:100
APC-eFluor780 anti-mouse c-Kit	eBioscience	Cat# 47-1171-82	1:100
Biotin anti-mouse c-Kit	BioLegend	Cat# 105804	1:100
PE-Cy7 anti-mouse Ter119	BioLegend	Cat# 116222	1:400
PE anti-mouse CD71	BioLegend	Cat# 113808	1:100
FITC anti-mouse CD41	BioLegend	Cat# 133903	1:100
FITC anti-mouse Ter119	eBioscience	Cat# 11-5921-82	1:400
FITC anti-mouse B220	BioLegend	Cat# 103206	1:400
FITC anti-mouse Gr-1	BioLegend	Cat# 108406	1:400
FITC anti-mouse CD2	BioLegend	Cat# 100105	1:400
FITC anti-mouse CD3	BioLegend	Cat# 100203	1:400
FITC anti-mouse CD8	BioLegend	Cat# 100706	1:400
PE anti-mouse CD41	BioLegend	Cat# 133906	1:100
PE anti-mouse Ter119	BioLegend	Cat# 116208	1:400
PE anti-mouse B220	BioLegend	Cat# 103208	1:400
PE anti-mouse Gr-1	BioLegend	Cat# 108408	1:400
PE anti-mouse CD2	BioLegend	Cat# 100108	1:400
PE anti-mouse CD3	BioLegend	Cat# 100308	1:400
PE anti-mouse CD8	BioLegend	Cat# 100708	1:400
PE anti-mouse Mac-1	BioLegend	Cat# 101208	1:400
PerCP/Cyanine5.5 anti-mouse CD41	BioLegend	Cat# 133918	1:100
PE-Cy7 anti-mouse CD16/32	BioLegend	Cat# 101318	1:100
PE-Cy5 anti-mouse Sca-1	BioLegend	Cat# 108110	1:100
PerCPCy5.5 anti-mouse B220	eBioscience	Cat# 45-0452-82	1:400
APC anti-mouse CD3	BioLegend	Cat# 100235	1:400
APC-eFluor780 anti-mouse Mac-1	eBioscience	Cat# 47-0112-82	1:400
PE-Cy7 anti-mouse Gr-1	eBioscience	Cat# 25-5931-82	1:400
APC-Cy7 anti-mouse CD45.1	BioLegend	Cat# 110716	1:100
FITC anti-mouse CD45.2	BioLegend	Cat# 109806	1:100
APC anti-BrdU	BD Biosciences	Cat# BDB557892	1:100
APC-Cy7 Streptavidin	BioLegend	Cat# 405208	1:200
APC anti mouse CD169 (Siglec-1)	BioLegend	Cat# 142418	1:200

FITC anti-mouse CD71	BioLegend	Cat# 113806	1:100
PE anti-mouse CD71	BioLegend	Cat# 113808	1:100
PerCP/Cyanine5.5 anti-mouse CD71	BioLegend	Cat# 113816	1:100
PE/Cyanine7 anti-mouse F4/80	BioLegend	Cat# 123114	1:200
PE/Cyanine7 anti-mouse CD127 (IL-7Rα)	BioLegend	Cat# 135014	1:100
PE/Cyanine5 anti-mouse CD135	BioLegend	Cat# 135312	1:100
PE/Cyanine7 anti-mouse CD105	BioLegend	Cat# 120410	1:100
APC anti-mouse CD34	BioLegend	Cat# 128612	1:50
PE/Cyanine7 anti-mouse CD150	BioLegend	Cat# 115914	1:100
PerCP/Cyanine5.5 anti-mouse Ly-6A/E (Sca-1)	BioLegend	Cat# 108124	1:100
CD16/CD32 anti-Mouse, Alexa Fluor™ 700	eBioscience	Cat# 50-168-73	1:100
Brilliant Violet 421™ anti-mouse CD3ε	BioLegend	Cat# 100341	1:400
Brilliant Violet 421™ anti-mouse TER-119	BioLegend	Cat# 116234	1:400
Brilliant Violet 421™ anti-mouse Ly-6G/Ly-6C (Gr-1)	BioLegend	Cat# 108445	1:400
Brilliant Violet 421™ anti-mouse CD8	BioLegend	Cat# 100753	1:400
Chemicals, Peptides, and Recombinant Proteins			
Polyinosinic-polycytidylic ribonucleic acid (Poly(I:C))	GE Healthcare	Cat# 27473201	
Tamoxifen	Sigma-Aldrich	Cat# T5648-1G	
5-Bromo-2′-deoxyuridine (BrdU)	Sigma-Aldrich	Cat# B5002	
Phenylhydrazine	Sigma-Aldrich	Cat# P26252-500G	
Deferoxamine mesylate salt	Sigma-Aldrich	Cat# D9533-1G	
Cycloheximide	Sigma-Aldrich	Cat# C7698	
Calpeptin	Sigma-Aldrich	Cat# C8999	
Z-DEVD-FMK	R&D systems	Cat# FMK004	
Ferrous Ammonium Sulfate, Hexahydrate	Sigma-Aldrich	Cat# FX0245-1	
Ascorbic acid	Sigma-Aldrich	A4544	
Critical Commercial Assays			
SuperScript VILO cDNA Synthesis Kit	Thermo Fisher Scientific	Cat# 11754250	
SYBR Premium Ex Taq (Tli RNaseH Plus), ROX Plus kit	Clontech	Cat# RR82WR	
BD APC BrdU Flow Kit	BD Biosciences	Cat# BDB557892	
RNeasy MinElute Cleanup Kit	QIAGEN	Cat# 74204	
Nextera XT DNA Library Prep Kit	Illumina	Cat# FC-131-1096	
KAPA Library Quantification Kit	KAPA Biosystems	Cat# KK4828	
NEBNext Ultra II DNA Library Prep Kit	New England BioLabs, Inc.	Cat# E7645	
EZ DNA Methylation-Direct Kit	Zymo research	Cat# D5020	
ZymoTaq PreMix	Zymo research	Cat# E2004	
Zymoclean Gel DNA Recovery Kits	Zymo research	Cat# D4008	
CloneJET PCR cloning kit	Thermo Fisher Scientific	Cat# K1231	

Deposited Data		
RNA-seq	This paper	GSE182059
scRNA-seq	This paper	GSE233845
Experimental Models: Organisms/Strains		
Mouse: Rosa26-LSL-tdTomato	The Jackson Laboratory	JAX: 007909
Mouse: Mx1-Cre	The Jackson Laboratory	JAX: 003556
Mouse: Tet2$^{fl/fl}$	The Jackson Laboratory	JAX: 017573
Mouse: R26-stop-EYFP	The Jackson Laboratory	JAX: 006148
Mouse: Ubc-GFP	The Jackson Laboratory	JAX: 004353
Mouse: Krt18-CreER	The Jackson Laboratory	JAX: 017948
Mouse: C57BL/6J	The Jackson Laboratory	JAX: 006494
Mouse: CD45.1	The Jackson Laboratory	JAX: 002014
Oligonucleotides		
Primer: Ermap Forward: CCTGCAGGTGGCAGTTTTAGG	This paper	N/A
Primer: Ermap Reverse: TGGGAACCATCCCACTGATTG	This paper	N/A
Primer: Slc4a1 Forward: CAAGCTCCGGTCAGGTCTAT	This paper	N/A
Primer: Slc4a1 Reverse: TCTCGAAGGTTTTCCTCCAGC	This paper	N/A
Primer: Car1 Forward: AGTGCAGTTAGTCATTTCACATATC	This paper	N/A
Primer: Car1 Reverse: CCATTGGTCAGGACCATTTTCG	This paper	N/A
Primer: Cldn13 Forward: AGCCAACAACGATACCTTAGATGT	This paper	N/A
Primer: Cldn13 Reverse: CCGCATCCAGAGTCCACTAC	This paper	N/A
Primer: Add2 Forward: ATAGCCACAGAGAAGCCTGGT	This paper	N/A
Primer: Add2 Reverse: ACTTCTGTCTTCTTGGTATCCTCTG	This paper	N/A
Primer: Dusp1 Forward: TATCGTGCCCAACGCTGAA	This paper	N/A
Primer: Dusp1 Reverse: ACGCTTCATATCCTCCTTGG	This paper	N/A
Primer: Fech Forward: TGGAGCACAATCGACAGGTG	Inokura et al., 2017	N/A
Primer: Fech Reverse: AACAGACATCGGCAGGGAGT	Inokura et al., 2017	N/A
Primer: Abcb7 Forward: TTACAAGATGTGAGCCTGGAAA	Inokura et al., 2017	N/A

Primer: Abcb7 Reverse: TTTGCGACTGCATATACTTCCTC	Inokura et al., 2017	N/A
Primer: Sf3b1 Forward: CAACACAGAATGGCTTTGGATA	Inokura et al., 2017	N/A
Primer: Sf3b1 Reverse: TCCTGTACTGCTCAGCTTCATC	Inokura et al., 2017	N/A
Primer: Slc25a38 Forward: GTGGTTCGCACAGAAAGTCTC	Inokura et al., 2017	N/A
Primer: Slc25a38 Reverse: GAAGAATACAGGGTGCCAAAGT	Inokura et al., 2017	N/A
Primer: Tfrc Forward: GAGGGTTATGTGGCATTCAGTA	Inokura et al., 2017	N/A
Primer: Tfrc Reverse: ATTTCCCCTGCTCTAACAATCA	Inokura et al., 2017	N/A
Primer: Slc25a37 Forward: CATGACAGCGGGAGCGAT	Inokura et al., 2017	N/A
Primer: Slc25a37 Reverse: GGCTTTGGGATCTGGATTCA	Inokura et al., 2017	N/A
Primer: Steap3 Forward: GCCAGTCTAACGCTGAGTACCT	Inokura et al., 2017	N/A
Primer: Steap3 Reverse: GCTTCTGGCTGATCACTGC	Inokura et al., 2017	N/A
Primer: Slc11a2 Forward: GAATCTGATTTGCAGTCTGGAG	Inokura et al., 2017	N/A
Primer: Slc11a2 Reverse: ACGGTGACATACTTCAGCAAGA	Inokura et al., 2017	N/A
Primer: Alas2 Forward: CCATCTTAAGGCAACCAAGGC	Inokura et al., 2017	N/A
Primer: Alas2 Reverse: ACAGCATGAAAGGACAATGGC	Inokura et al., 2017	N/A
Primer: Tfrc Forward: GTATAATTTGGATAAAGAAGGGTTTAAA	This paper	N/A
Primer: Tfrc Reverse: TCTATACTTTACTACCCAACCTATAATA	This paper	N/A
NEBNext® Multiplex Oligos for Illumina® (Dual Index Primers Set 1)	New England BioLabs, Inc.	Cat# E7600S
