# Supplementary material for: Increased iron uptake by splenic hematopoietic stem cells promotes TET2-dependent erythroid regeneration
Source: Nat Commun. 2024 Jan 15;15:538. doi: 10.1038/s41467-024-44718-0 (PMC10789814; doi:10.1038/s41467-024-44718-0)
Supplement: Supplementary file 3 — Reporting Summary [file 41467_2024_44718_MOESM3_ESM.pdf]

nature portfolio

Corresponding author(s): Daisuke Nakada

Last updated by author(s): Dec 7, 2023

Reporting Summary

Nature Portfolio wishes to improve the reproducibility of the work that we publish. This form provides structure for consistency and transparency in reporting. For further information on Nature Portfolio policies, see our Editorial Policies and the Editorial Policy Checklist.

Statistics

For all statistical analyses, confirm that the following items are present in the figure legend, table legend, main text, or Methods section.

n/a	Confirmed	
☐	☒	The exact sample size (*n*) for each experimental group/condition, given as a discrete number and unit of measurement
☐	☒	A statement on whether measurements were taken from distinct samples or whether the same sample was measured repeatedly
☐	☒	The statistical test(s) used AND whether they are one- or two-sided<br>*Only common tests should be described solely by name; describe more complex techniques in the Methods section.*
☐	☒	A description of all covariates tested
☐	☒	A description of any assumptions or corrections, such as tests of normality and adjustment for multiple comparisons
☐	☒	A full description of the statistical parameters including central tendency (e.g. means) or other basic estimates (e.g. regression coefficient) AND variation (e.g. standard deviation) or associated estimates of uncertainty (e.g. confidence intervals)
☐	☒	For null hypothesis testing, the test statistic (e.g. *F*, *t*, *r*) with confidence intervals, effect sizes, degrees of freedom and *P* value noted<br>*Give P values as exact values whenever suitable.*
☒	☐	For Bayesian analysis, information on the choice of priors and Markov chain Monte Carlo settings
☒	☐	For hierarchical and complex designs, identification of the appropriate level for tests and full reporting of outcomes
☒	☐	Estimates of effect sizes (e.g. Cohen's *d*, Pearson's *r*), indicating how they were calculated

Our web collection on statistics for biologists contains articles on many of the points above.

Software and code

Policy information about availability of computer code

Data collection	For RNA sequencing, STAR (version 2.5.2b) was used to align mRNA reads to mm10.<br>For single cell RNA sequencing, Cell Ranger v3 was used to align mRNA reads to mm10.
Data analysis	All analysis was performed using free, open-source packages such as DESeq2 (version 1.12.4), Seurat v4, and Bismark (v0.22.2), or with softwares such as GraphPad Prism 8.4.3 as ditailed in Methods.

For manuscripts utilizing custom algorithms or software that are central to the research but not yet described in published literature, software must be made available to editors and reviewers. We strongly encourage code deposition in a community repository (e.g. GitHub). See the Nature Portfolio guidelines for submitting code & software for further information.

Data

Policy information about availability of data

All manuscripts must include a data availability statement. This statement should provide the following information, where applicable:

- Accession codes, unique identifiers, or web links for publicly available datasets
- A description of any restrictions on data availability
- For clinical datasets or third party data, please ensure that the statement adheres to our policy

The RNA sequencing and bisulfite sequencing dataset generated in this study are in the Gene Expression Omnibus (GEO) under the accession numbers (GSE182059 and GSE233845).

Research involving human participants, their data, or biological material

Policy information about studies with human participants or human data. See also policy information about sex, gender (identity/presentation), and sexual orientation and race, ethnicity and racism.

Reporting on sex and gender	No human participants in this study.
Reporting on race, ethnicity, or other socially relevant groupings	N/A
Population characteristics	N/A
Recruitment	N/A
Ethics oversight	N/A

Note that full information on the approval of the study protocol must also be provided in the manuscript.

Field-specific reporting

Please select the one below that is the best fit for your research. If you are not sure, read the appropriate sections before making your selection.

- [x] Life sciences
- [ ] Behavioural & social sciences
- [ ] Ecological, evolutionary & environmental sciences

For a reference copy of the document with all sections, see nature.com/documents/nr-reporting-summary-flat.pdf

Life sciences study design

All studies must disclose on these points even when the disclosure is negative.

Sample size	The majority of experiments were repeated at least three times to obtain data for indicated statistical analyses. Mice were allocated to experimental groups based on their genotype and randomized within the designated sex- and age-matched groups. Given that our mice were inbred and matched for age and sex, we consistently assumed similar variance between the different experimental groups. Sample sizes were chosen based on observed effect sizes and standard errors from previous experiments.
Data exclusions	No data were excluded.
Replication	The majority of experiments were repeated at least three times to ensure reproducibility. Statistical analyses were conducted to illustrate significance, and all attempts to replicate experiments were successful.
Randomization	Mice were allocated to experimental groups on the basis of their genotype and randomized within the given sex- and age-matched group.
Blinding	No blinding was performed.

Reporting for specific materials, systems and methods

We require information from authors about some types of materials, experimental systems and methods used in many studies. Here, indicate whether each material, system or method listed is relevant to your study. If you are not sure if a list item applies to your research, read the appropriate section before selecting a response.

Materials & experimental systems

n/a	Involved in the study	
[ ]	[x]	Antibodies
[x]	[ ]	Eukaryotic cell lines
[x]	[ ]	Palaeontology and archaeology
[ ]	[x]	Animals and other organisms
[x]	[ ]	Clinical data
[x]	[ ]	Dual use research of concern
[x]	[ ]	Plants

Methods

n/a	Involved in the study	
[x]	[ ]	ChIP-seq
[ ]	[x]	Flow cytometry
[x]	[ ]	MRI-based neuroimaging

Antibodies

Antibodies used	TET2 (D6C7K) Rabbit mAb (Mouse Specific) Cell-Signaling Technology Cat# 36449S 1:500 or 1:1000<br>DyLight™ 554 Phalloidin Cell-Signaling Technology Cat# 13054S 1:200

Goat anti-Rabbit IgG Secondary Antibody, Alexa Fluor 488 Invitrogen Cat# A-11008 1:500
PE-Cy5 anti-mouse CD150 (TC15-12F12.2) BioLegend Cat# 115912 1:100
PE anti-mouse CD48 (HM48-1) BioLegend Cat# 103406 1:100
PE-Cy7 anti-mouse CD48 (HM48-1) BioLegend Cat# 103424 1:100
APC anti-mouse Sca-1 (D7) eBioscience Cat# 17-5981-82 1:100
APC-eFluor780 anti-mouse c-Kit (2B8) eBioscience Cat# 47-1171-82 1:100
Biotin anti-mouse c-Kit BioLegend (2B8) Cat# 105804 1:100
PE-Cy7 anti-mouse Ter119 (TER-119) BioLegend Cat# 116222 1:400
PE anti-mouse CD71 (RI7217) BioLegend Cat# 113808 1:100
FITC anti-mouse CD41 (MWReg30) BioLegend Cat# 133903 1:100
FITC anti-mouse Ter119 (TER-119) eBioscience Cat# 11-5921-82 1:400
FITC anti-mouse B220 (RA3-6B2) BioLegend Cat# 103206 1:400
FITC anti-mouse Gr-1 (RB6-8C5) BioLegend Cat# 108406 1:400
FITC anti-mouse CD2 (RM2-5) BioLegend Cat# 100105 1:400
FITC anti-mouse CD3 (17A2) BioLegend Cat# 100203 1:400
FITC anti-mouse CD8 (53-6.7) BioLegend Cat# 100706 1:400
PE anti-mouse CD41 (MWReg30) BioLegend Cat# 133906 1:100
PE anti-mouse Ter119 (TER-119) BioLegend Cat# 116208 1:400
PE anti-mouse B220 (RA3-6B2) BioLegend Cat# 103208 1:400
PE anti-mouse Gr-1 (RB6-8C5) BioLegend Cat# 108408 1:400
PE anti-mouse CD2 (RM2-5) BioLegend Cat# 100108 1:400
PE anti-mouse CD3 (17A2) BioLegend Cat# 100308 1:400
PE anti-mouse CD8 (53-6.7) BioLegend Cat# 100708 1:400
PE anti-mouse Mac-1 (M1/70) BioLegend Cat# 101208 1:400
PerCP/Cyanine5.5 anti-mouse CD41 (MWReg30) BioLegend Cat# 133918 1:100
PE-Cy7 anti-mouse CD16/32 (93) BioLegend Cat# 101318 1:100
PE-Cy5 anti-mouse Sca-1 (D7) BioLegend Cat# 108110 1:100
PerCPCy5.5 anti-mouse B220 (RA3-6B2) eBioscience Cat# 45-0452-82 1:400
APC anti-mouse CD3 (17A2) BioLegend Cat# 100235 1:400
APC-eFluor780 anti-mouse Mac-1 (M1/70) eBioscience Cat# 47-0112-82 1:400
PE-Cy7 anti-mouse Gr-1 (RB6-8C5) eBioscience Cat# 25-5931-82 1:400
APC-Cy7 anti-mouse CD45.1 (A20) BioLegend Cat# 110716 1:100
FITC anti-mouse CD45.2 (104) BioLegend Cat# 109806 1:100
APC anti-BrdU BD Biosciences Cat# BDB557892 1:100
APC-Cy7 Streptavidin BioLegend Cat# 405208 1:200
APC anti mouse CD169 (Siglec-1, 3D6.112) BioLegend Cat# 142418 1:200
FITC anti-mouse CD71 BioLegend Cat# 113806 1:100
PE anti-mouse CD71 (RI7217) BioLegend Cat# 113808 1:100
PerCP/Cyanine5.5 anti-mouse CD71 (RI7217) BioLegend Cat# 113816 1:100
PE/Cyanine7 anti-mouse F4/80 (BM8) BioLegend Cat# 123114 1:200
PE/Cyanine7 anti-mouse CD127 (IL-7Rα, A7R34) BioLegend Cat# 135014 1:100
PE/Cyanine5 anti-mouse CD135 (A2F10) BioLegend Cat# 135312 1:100
PE/Cyanine7 anti-mouse CD105 (MJ7/18) BioLegend Cat# 120410 1:100
APC anti-mouse CD34 (HM34) BioLegend Cat# 128612 1:50
PE/Cyanine7 anti-mouse CD150 (TC15-12F12.2) BioLegend Cat# 115914 1:100
PerCP/Cyanine5.5 anti-mouse Ly-6A/E (Sca-1, D7) BioLegend Cat# 108124 1:100
CD16/CD32 anti-Mouse (93), Alexa Fluor™ 700 eBioscience Cat# 50-168-73 1:100
Brilliant Violet 421™ anti-mouse CD3ε (145-2C11) BioLegend Cat# 100341 1:400
Brilliant Violet 421™ anti-mouse TER-119 (TER-119) BioLegend Cat# 116234 1:400
Brilliant Violet 421™ anti-mouse Ly-6G/Ly-6C (Gr-1) BioLegend Cat# 108445 1:400
Brilliant Violet 421™ anti-mouse CD8 (53-6.7) BioLegend Cat# 100753 1:400

Validation

Antibodies for flow cytometry was validated with according to manufacturers' instruction. Fluorescence minus one controls were used when new antibodies/batch were obtained. Antibodies for immunoblotting was validated with knockout cells whenever available.

Animals and other research organisms

Policy information about studies involving animals; ARRIVE guidelines recommended for reporting animal research, and Sex and Gender in Research

Laboratory animals

Mouse: Rosa26-LSL-tdTomato The Jackson Laboratory JAX: 007909
Mouse: Mx1-Cre The Jackson Laboratory JAX: 003556
Mouse: Tet2fl/fl The Jackson Laboratory JAX: 017573
Mouse: R26-stop-EYFP The Jackson Laboratory JAX: 006148
Mouse: Ubc-GFP The Jackson Laboratory JAX: 004353
Mouse: Krt18-CreER The Jackson Laboratory JAX: 017948

	Mouse: C57BL/6J The Jackson Laboratory JAX: 006494<br>Mouse: CD45.1 The Jackson Laboratory JAX: 002014<br><br>Mice were housed in AAALAC-accredited, specific-pathogen-free animal care facilities at Baylor College of Medicine (BCM) with 12-hour light-dark cycle, ambient temperature at 70 degrees Fahrenheit, humidity within 30%-80%, and received standard chow ad libitum. Mice 8 to 12 weeks of age of both sexes were used, and experimental mice were separated by sex and housed with up to five mice per cage.
Wild animals	N/A
Reporting on sex	Mice of both sexes were used in the study with no sex differences.
Field-collected samples	N/A
Ethics oversight	All procedures were approved by the BCM (protocol #AN-5858) Institutional Animal Care and Use Committees.

Note that full information on the approval of the study protocol must also be provided in the manuscript.

Plants

Seed stocks	N/A
Novel plant genotypes	N/A
Authentication	N/A

Flow Cytometry

Plots

Confirm that:

- [x] The axis labels state the marker and fluorochrome used (e.g. CD4-FITC).
- [x] The axis scales are clearly visible. Include numbers along axes only for bottom left plot of group (a 'group' is an analysis of identical markers).
- [x] All plots are contour plots with outliers or pseudocolor plots.
- [x] A numerical value for number of cells or percentage (with statistics) is provided.

Methodology

Sample preparation	Bone marrow cells were either flushed from the long bones (tibias and femurs) or isolated by crushing the long bones (tibias and femurs), pelvic bones, vertebrae, and sternum with mortar and pestle in Hank's buffered salt solution (HBSS) without calcium and magnesium, supplemented with 2% heat-inactivated bovine serum (GIBCO, Grand Island, NY). Splenocytes were dissociated by smashing the entire spleen between slides. Cells were triturated and filtered through a nylon screen (100 µm, Sefar America, Kansas City, MO) or a 40 µm cell strainer (Fisher Scientific, Pittsburg, PA) to obtain a single-cell suspension.
Instrument	Flow cytometry was performed with FACSAria II, FACSCanto II, LSR II, or LSRFortessa flow-cytometers (BD Biosciences).
Software	FACS Diva, Flowjo
Cell population abundance	Post sort verification of HSCs and LSK cells were determined to be more than 95% pure.
Gating strategy	For hematopoietic lineage tracing, 17 populations were analyzed along with HSCs: MPPs (LSKCD150-CD48-/low), HPC2 (LSKCD150+CD48+), HPC1 (LSKCD150-CD48+), GMP (Lin-Sca1-c-kit+CD34+CD16/32+), CMP (Lin-Sca1-c-kit+CD34+CD16/32-/low), MEP (Lin-Sca1-c-kit+CD34-/lowCD16/32-/low), CLP (Lin-Sca1lowc-kitlowCD127+CD135+), MkP (Lin-Sca1-c-kit+CD150+CD41+), pre CFU-E (Lin-Sca1-c-kit+CD41-CD16/32-CD150+CD105+), pre MegE (Lin-Sca1-c-kit+CD41-CD16/32-CD150+CD105-/low), pre GM (Lin-Sca1-c-kit+CD41-CD16/32-CD150-CD105-), ProE (CD71+Ter119medium)81, EryA (Ter119highCD71+FSChigh), EryB (Ter119highCD71+FSClow), EryC (Ter119highCD71-/lowFSClow), and lineage+ cells (CD3+, B220+, Mac1/Gr1+, CD41+, and Ter119+), CFU-E (Lin-Sca1-c-kit+CD41-CD16/32-CD150-/lowCD105+CD71+Ter119-/low)83, stress erythroid progenitor population I (c-kit+CD71low/medTer119low/–), population II (c-kit+CD71hiTer119med), and population III (c-kit+CD71low/medTer119high). Positive and negative gates were based on prior publication or FMO controls.

- [x] Tick this box to confirm that a figure exemplifying the gating strategy is provided in the Supplementary Information.
